# 109 years of forest growth measurements from individual Norway spruce trees

**DOI:** 10.1038/sdata.2018.77

**Published:** 2018-04-24

**Authors:** Georg E. Kindermann, Ferdinand Kristöfel, Markus Neumann, Günter Rössler, Thomas Ledermann, Silvio Schueler

**Affiliations:** 1 Austrian Research Center for Forests. Seckendorff-Gudent-Weg 8, 1131 Vienna, Austria

**Keywords:** Forest ecology, Forestry

## Abstract

In 1892 a forest spacing experiment with four different spacing patterns was established with Norway spruce (*Picea abies*). From 1923 until 1997, when the stand was harvested, diameter, height and height to crown base of in total 4507 trees were measured up to 23 times. The original aim of the experiment during establishment was to analyse short term effects of different spacing patterns. The thinning regime followed state of the art forestry practises. During the observation time of more than 100 years, the individual observers and the measurement technology changed several times. Thus, the raw measurement data contain systematic and unsystematic measurement errors as well as missing data. We developed methods to complete missing data, smoothen implausible developments, and correct measurement errors. The data provided in the present study include spatially explicit individual-tree growth data which can be used to analyse the development of forest stands and its individual trees during one rotation period. The data can be used e.g. to parameterize and validate forest growth and competition models.

## Background & Summary

In 1892, the Austrian Research Center for Forests established a spacing experiment with Norway spruce (*Picea abies* [L.] Karst.) in the Vienna Woods. The so called ‘Hauersteig’ experiment (Lat=48.2254°, Lon=16.1353°) comprised four plots with the following planting distances: 1×1 m, 1.5×1.5 m, 1×2 m and 2×2 m. The measurements of individual-tree growth started in 1923 at a stand age of 35 years. In 1997 at the age of 109 years, a final survey was conducted before harvesting the remaining trees. The first measurement included 4507 trees, 456 of which survived until 1997. Density reductions were implemented following common management practices. Overall, plots and individual trees were measured 23 times within intervals of 1 to 6 years. Typical measures include diameter at 1.3 m height, tree height and height of the living crown. Additional stem disc analyses of harvested trees allowed analyzing diameter growth along the tree trunk. During this time period, the measurement equipment had changed. In the early years, the diameter was measured crosswise with a caliper. Later on the girth was measured with a tapeline. During the early phase of the experiment, the height was measured with bamboo poles, then with ladder and tapeline. Later clinometers from different manufacturers and with different ways of measurement was used to evaluate the required distance to the tree. Discrepancies due to different observers and measuring equipment were reduced by comparing the repeated diameter and height measurements with the diameters and height from the stem analyses. Observation gaps were filled up by interpolation between early and later observations of the tree. Missing values were complemented by calculating averages of comparable measured trees. Finally, diameter and height were proved to have increased monotonically, except that a crown breakage was recorded. The provided spatially explicit individual-tree growth data can be used to analyse the development of individual trees and forest stands during one rotation period. It can be used to parameterize and validate forest growth models or compare different competition indices.

## Methods

The experiment was established in spring 1892 using three year old seedlings of Norway spruce by applying the pit-planting method. The provenance was not recorded. To avert game damages, the trial was fenced in the year of establishment. In total, the trial had a size of 1.9 ha (160.4 m×118.4 m) and was split up into four plots (Table 1). The density within each of these four plots ranged from 2500 trees/ha up to 10000 trees/ha (Table 1).

The very little juvenile mortality was redeemed by reinforcement planting over the first years to maintain the original spacing pattern. Until 1923 the plots had an undisturbed growth and only few plot observations had been done. The canopy closure – the crowns of neighbour trees are getting into contact-was reached in 1898 on plot 1, between 1901 and 1902 on plot 2, on plot 3 in 1899 in the 1 m direction and in 1903 in the 2 m direction, and in 1905 on plot 4. Beside the planted Norway spruce few trees of European larch (*Larix decidua*), Scots pine (*Pinus sylvestris*), European beech (*Fagus sylvatica*), sessile oak (*Quercus petreae*) and silver birch (*Betula pendula*) regenerated naturally on the trial.

In 1923 an observation area with a size of approximately 0.25 hectare was permanently established on each of the four plots. These core zones were surrounded by a buffer zone. The trees on these core zones were marked permanently with an individual number in the year 1923. On plot 1 only the elite trees got an individual tree number in 1923, the remaining trees on plot 1 where marked in 1925. The trees on plot 1 which had been removed between 1923 and 1925 were measured but had not been provided with individual tree numbers.

### Measurements

Throughout the observation time different measurement techniques were used for assessing tree height and diameter of standing trees (Table 2). From 1923 until 1973 the diameter at 1.3 m tree height (D1.3) was measured crosswise with a calliper, and from 1978 onwards via girth using a tapeline. Tree height of standing trees was mostly measured on subsamples. The sampling was either systematic or related to the 100 thickest or done on harvested trees. Tree heights of the young stand were measured with bamboo poles; later measurements used ladder and tapeline, Blume-Leiss hypsometer with fixed distance, Blume-Leiss hypsometer with variable distance after^1^, Blume-Leiss hypsometer with variable distance measured with tapeline, Clinometer (Enbeeco Teleclino 7×50), and finally with Vertex (Haglöf Forestor Vertex II).

From all trees which had been harvested before 1970 the diameter was measured along the trunk regularly at intervals of 1 m distance. Also tree height of the harvested trees was measured with a tapeline. In addition, stem analyses were made from selected felled trees. For such stem analyses, stem discs were cut at specified tree heights and the number and width of the tree rings were measured from these discs. Thus, the height and diameter growth inside bark could be reconstructed.

**Figure 1:**
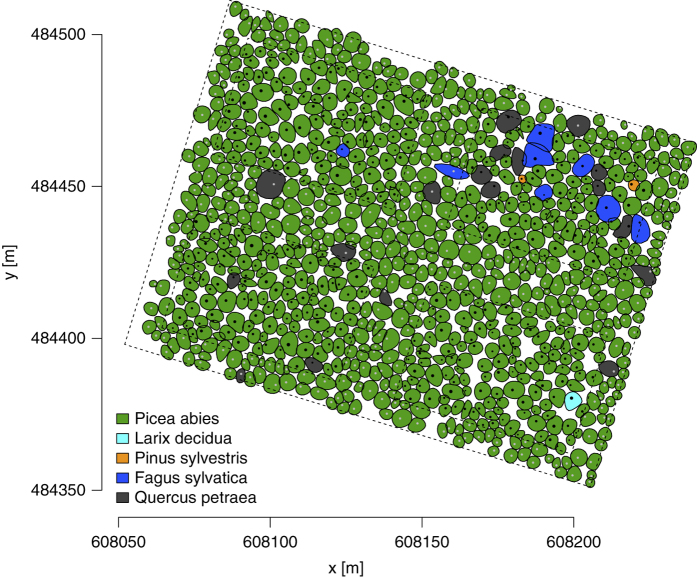
Crown map of the whole plot in 1988. Map projection EPSG:31287 MGI Austria Lambert.

In winter 1988, the coordinates of the tree base position were measured and used to draw a map of tree positions and the corresponding crown shapes. Using this map the crown radii in 8 directions were measured and recorded (see Fig. 1). In other years only one crown diameter per tree was measured.

### Spatial coordinates

**Figure 2:**
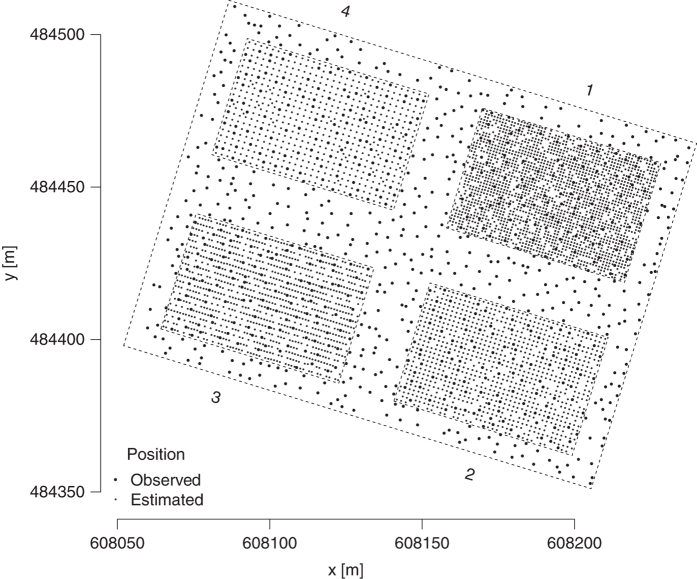
Map of tree positions. Map projection EPSG:31287 MGI Austria Lambert.

In 1988 the exact spatial locations of all standing trees were mapped. However, trees that had been removed before 1988 had no exact coordinates. Therefore, the coordinates of these trees were reconstructed on the basis of their tree number. In order to obtain a full spatial distribution of both removed and standing trees, we compared the actual tree positions (in 1988) and the tree numbering system established 1923/25 to identify possible positions of harvested trees. The positions of removed trees were found to be distributed in wavy lines on the individual plots. Positions of trees which had a number beyond this pattern, which was mainly the case on plot 1 for trees removed already in 1923, were placed randomly on the raster. With the tree positions in 1988, the original spacing pattern was reconstructed by adjusting the inter-tree distances until the differences between the measured positions and the raster positions reached a minimum. Figure 2 shows the positions of all trees which had been observed in 1923. The restored planting patterns were as follows: 1.002 m by 1.000 m on plot 1, 1.490 m by 1.500 m on plot 2, 1.000 m by 1.980 m on plot 3 and 2.010 m by 1.993 m on plot 4. As the plot corners had also been measured, we tested if they were aligned exactly between the planting rows. If this was not the case an auxiliary corner was placed exactly between the planting rows. These adjusted plot borders were used for calculating values related to the plot area. The altitude of each tree was obtained from using its coordinates, using a high-resolution (1 m×1 m) digital elevation map.

### Diameter adjustments for measuring error

Throughout the lifetime of the trial, management and measurement were carried out by different observers using different measurement techniques (Table 2). These factors might have caused a bias in the obtained tree parameters. As for a sample of trees, stem discs at tree height of 1.3 m tree height have been harvested. We utilized stem disc analyses to compare the various diameter measurements during different observation years and calculated adjusted diameter values. This comparison was based on the following assumptions: 1) when diameter was measured during the vegetation period, we assumed that diameter increment followed a linear increase between the May 1 and August 31, which is close to the observations of (ref. 2). 2) Diameter assessment was either done by one or two cross sectional measurements or by measuring the girth, whereas during the stem analysis four radii from the centre in direction North, East, South and West were measured. The arithmetic mean of diameters was used to average cross-sectional measurements as it revealed comparable results as with the theoretical better geometric mean^3^. Orthogonal measured stem disc radii were averaged according to ref. 4 who showed that the arithmetic mean of the basal areas of the individual radii comes close to the real basal area of the stem.

To compare tree diameter from stem discs with the periodic measurements on living trees, we had to correct them for the missing tree bark and shrinkage after tree harvest and wood drying. The relative diameter differences (dm – dd)/dd of stem disc diameter (dd) and repeated measurements (dm) were plotted over stem disc diameter in order to obtain bias correction separately for 1) all diameter measures with a calliper and 2) diameter measures with tape. In relation to the stem disc diameter, the diameter of unshrinked wood outside bark measured with calliper was found to be 7.0% larger and the diameter of unshrinked wood outside bark measured with tape was found to be 8.2% larger. The difference of 1.2% between calliper and tape measurement is likely caused by non-circular stems. Finally, the diameters of stem disc measurements have been increased by 7.0% to represent stem diameters of unshrinked wood outside bark. Now this adjusted stem disc diameter can be compared with the diameter from the repeated measurements and periodic fluctuations due to measurement error could be eliminated. These periodic fluctuations can be handled by (1) eliminating the absolute diameter difference, (2) the relative diameter difference or (3) the relative basal area difference. We choose the basal area to reduce differences by eliminating the median of the relative basal area differences for each year and subplot. After this diameter adjustment, outliers causing diameter increments lower than −1 cm and −0.54 cm/year respectively and larger than 1 cm/year were removed if there are enough other measurements. Negative diameter increments have been eliminated by computing an isotonic (monotone increasing nonparametric) least squares regression which is piecewise constant (isoreg).

To correct for missing values, i.e. individual trees that had not been measured during a regular field campaign, a list of observation dates was build for each plot and each individual tree. A missing tree observation was recorded when a plot observation date had no tree observation and occurred before the last tree observation. Diameters for such missing values were estimated by a spline function using the method “monoH.FC” by interpolating between the basal area of previous and subsequent measurement of the individual trees. After this complement it was assured that also these trees had a monotonically increasing diameter. Periods with no diameter increment were eliminated if possible by linear interpolation between previous and subsequent observation.

### Height adjustment

Tree heights of young trees and removed trees had been measured directly either by using poles or ladders and tapeline. The height of larger trees had been calculated by triangulation using observer-tree distances and angles between tree basis and tree top. These height calculations had been done either implicitly, i.e. the tree height was read directly from the height measurement instrument, or the measured distances and angles had been recorded and the height calculations had been done in a second step at the office. In the latter case, measurement data had been recorded in fixed format (3 characters, angle given as decimal degree) on punch cards. Negative angles (direction downslope) had been coded by setting the first digit to 9. Post-measurement calculations of tree height had been done for measurements in the years 1978, 1983, 1989 and 1993. In 1978, the angles to the tree top and bases had been measured and the variable distance from the observer to the tree had been measured after^1^. In 1983, 1989 and 1993 the slope distance observer-tree distance and angles to the top and bottom of the tree had been measured. The heights in 1978 could be calculated with

$${H=A*C*\cos}^{2}\left(\mathrm{WU}\right)*\left[\tan\left(\mathrm{WO}\right)+\tan\left(\mathrm{WU}\right)\right]+K$$

where *H* is the height in dm, *A* the distance of the measurements marks of the tree in cm, *C* a device dependent constant (in this case 1/0.3), WO the angel to the tree top in °, WU the angle to the measurement mark on the stem in ° and K the height of the measurement mark in dm (typical 13 dm above ground).

The heights of 1983, 1989 and 1993 could be calculated with

H=D*cos(WU)*[tan(WO)+tan(WU)]+K

where *D* is the slope distance between clinometer and measurement mark on the stem in dm.

Tree height measurements could contain certain biases due to changing measurement technologies, varying observation positions for individual trees if the trees are not exactly perpendicular, and the difficulty to identify the exact tree top especially for non- monopodial trees. In order to estimate the measurement bias and to remove implausible repeated measurements (i.e. decreasing tree heights in subsequent observations), we corrected height data with several logical verifications. In a first step we tested for negative height increments larger than 1 m. If such trees did not show signs of crown breaks or other crown damages, negative height increments were considered as measurement bias and removed from the dataset. In the second step we tested for extreme positive height increments of more than 1 m annually. Such values are implausible under the given site conditions and were also removed.

For the remaining heights, an adjustment procedure on basis of stem disc analysis was applied similarly as for tree diameter. As stem discs have been collected in different heights, counting of tree rings allowed identifying the tree age of the respective disc height. It was assumed that this stem disc height was reached in the middle of the vegetation period and thus the counted age was reduced by a half year. Tree heights of ages between stem discs were estimated by using a spline with the method "monoH.FC”. The median of the relation between the height from the stem analysis/height from repeated measurement for each plot and observation year was levelled out by multiplying the repeated measured height with a plot and year specific factor.

Then negative height increments of this adjusted tree heights were eliminated in cases where no crown break was recorded by isoreg and named hMon. Trees without any crown break that reached the final stand age of 109 years in 1997 were used to develop a height age curve in the form h=c0*log(1+exp(c2)*age**c3)**c1 according to ref. 5. This function was used to estimate tree height (hLogFun) of every tree in dependence of its age. The relation of hMon/hLogFun was fitted with a Generalized Additive Mixed Model (GAMM) by using a spline over age, diameter, x- and y-position for spruce and using only age and diameter for the other species by selecting the individual tree or species for applying a random effect on the intercept. With this GAMM the height of each tree (without a crown break) for each observation period was estimated. Negative height increments due to spline fittings were eliminated. Now these calculated heights are corrected with a multiplier so that they hit the - bias corrected and monotonic increasing-measured heights. This was done by linear interpolating the relation of these two heights between the observation dates. This fit was done once with all height measurements the other time only until the age when the crown break was recorded. The later height (height estimate without crown break even if there was a crown break recorded) was used in the next step for estimating the height of the crown base. Negative height increments of this height are eliminated, as long as there was no crown break and zero increments have been tried to be removed by using the average date of this constant height and interpolate the height for the observation dates linear by using the previous and next observation.

The measured height of the crown base needs to be lower or equal than the tree height. As spruce cannot expand its crown downwards the observed crown heights are brought to monotone increasing values with isoreg. With a GAMM the height of the crown base and also its ratio (height of the crown base / tree height) were estimated by using a spline over age, diameter, estimated height without a crown break, x- and y-position by selecting the single tree and if this is not possible, due to too few observations, the species for applying a random effect on the intercept. From these two crown height estimates (direct estimate of height of the crown base and based on the crown ratio estimate) the average was calculated. This average was brought to monotonically increasing values with isoreg. The crown heights were adjusted to hit the monotonically increasing observations and were checked again, if those heights were still monotonically increasing. Zero height changes were tried to be removed by linear interpolateing the crown height for the observation dates in between by using the previous and next observation. Finally it was tested if the crown base was not higher than the tree height.

### Code availability

The code processing the data is included in the dataset and has the file name “dhc.r”.

## Data Records

The data is provided in comma separated values format (Data Citation 1).

corner.csv: Holds the positions of the plot corners

plot: Plot number which had different spacing patterns (1...1x1 m, 2...1.5x1.5 m, 3...1x2 m, 4...2x2 m)

corner: Corner number

cor: Position corrected (TRUE) or position measured in forest (FALSE)

x: x-Coordinate in m, projection EPSG:31287 MGI Austria Lambert

y: y-Coordinate in m

lon: Longitude in °

lat: Latitude in °

 

area.csv: Contains the plot area

plot: Plot number

area: Plot are in m^2^

 

pos.csv: Contains tree species, its position, if it is in the core area, the germination year, removal date and removal reason

plot: Plot number

tree: Tree number

x: x-Coordinate in m

y: y-Coordinate in m

z: Altitude in m

species: Species code following EN 13556. PCAB...Picea abies, LADC...Larix decidua, PNSY...Pinus sylvestris, FASY...Fagus Sylvatica, QCPE...Quercus petraea, BTPE...Betula pendula

posObs: Indicating if the position is measured (TRUE) or estimated (FALSE)

germinationYear: Giving the germination year which is 1888 for spruce and for all other species it was estimated in 1892.

core: Tree is in core area (TRUE) or in the surrounding arrea (FALSE)

removeDate: Date of removal Year-Month-Day

removeal: Reason of removal. 1...planed cut, 2...snow break, 3...wind throw or windbreak, 4...other abiothic damages, 5...insects, 6...fungus, 7...bark peeling, 8...tree missing, 9...competition

lon: Longitude in °

lat: Latitude in °

 

date.csv: Observation date

plot: Plot number

year: Observation year

obs: Observation in this year

date: Observation date Year-Month-Day

 

dbhObs.csv: Repeated measurements of the single tree

plot: Plot number

tree: Tree number

year: Observation year

obs: Observation in this year

dbh: Diameter in 1.3 m tree height in mm

dbh2: Orthogonal measured second diameter in mm

hmk: Selection criteria to measure tree height. 1...systematic, 2...systematic and in the group of the 100 thickest, 3...belongs to the 100 thickest, 4...lying tree, 5...Standing tree with a ladder, 6...outlier, 7...from stemanalysis

kh: Type of the height measurement. 0...tree height, 1...angle and distances

ho: Tree height in dm when kh=0. When kh=1 then distance to the tree in dm or in 1977 length of the base bar in cm

ka: Height to the crown base in dm when kh=0. When kh=1 then angle to the tree top in 1/10 degree.

kb: Crown width in dm when kh=0. When kh=1 then angle to 1.3 m above tree base in 1/10 degree.

wka: Angle to crown base in 1/10 degree.

kraft: Tree classes according to Kraft. 1...predominant, 2...dominant, 3...co-dominant, 4...dominated, 5...overtopped

crown: Crown quality. 0...normal, 1...broken in the crown region, 2...substituted tree top, 3...forked, 4...bushy, stork nest, witches' broom, 5...wizen tree top, 6...again broken tree top

stem: Stem quality. 0...typical, 1...crooked, 2...abiotic damaged, 3...biotic damaged, 4...forked stem without damage, 5...forked stem with damage, 6...up to 1/3 of the girth is peeled, 7...more than 1/3 of the girth is peeled, 8...broken stem, 9...other stem damages

defoliation: crown defoliation. 1...low, 2...medium, 3...much

 

crown.csv: Measured crown radii

plot: Plot number

tree: Tree number

year: Observation year

obs: Observation in this year

d17: Crown radius in direction 17° from the stem in m

d62: Crown radius in direction 62° from the stem in m

d107: Crown radius in direction 107° from the stem in m

d152: Crown radius in direction 152° from the stem in m

d197: Crown radius in direction 197° from the stem in m

d242: Crown radius in direction 242° from the stem in m

d287: Crown radius in direction 287° from the stem in m

d332: Crown radius in direction 332° from the stem in m

 

secDiam.csv: Stem diameter measured along the trunk

plot: Plot number

tree: Tree number

year: Observation year

species: Species code following EN 13556. PCAB...Picea abies, QCPE...Quercus petraea

length: Stem length in m

dbh: Diameter in 1.3 m height in cm

crownLength: Length of the crown in m

crownWidth: With of the crown in m

m1 – m27: Diameter at height 1 m to 27 m in cm

 

saTree.csv: Tree information for stem analysis

plot: Plot number

tree: Tree number

species: Species code following EN 13556. PCAB...Picea abies, PNSY...Pinus sylvestris

dbh: Diameter in 1.3 m height in cm

height: Tree height in m

hKrown: Height of the crown base m

 

saIr.csv: Ring width from stem analysis

plot: Plot number

tree: Tree number

disc: Stem disc number

dir: Measurement direction

year: Year of radial increment

ir: Radial increment in 1/100 mm

 

saDisc.csv: Stem disc information for stem analysis

plot: Plot number

tree: Tree number

disc: Stem disc number

h: Height of the disc in m

 

dhcComplSmooth.csv: Processed diameter, height and crown base where observation gaps are filled and values are monotone increasing using the processing stepsgiven in the file “dhc.r”.

plot: Plot number

tree: Tree number

year: Observation year

obs: Observation in this year

d: Diameter in 1.3 m tree height in mm

h: Tree height

hCr: Height of the crown base

## Technical Validation

The precision of measurement instruments throughout the observation time changed, but today we cannot precisely judge the quality and observation scale of the instruments used at the beginning of the experiment. Therefore, we tested if the reading of the diameter measurement had an accuracy of 1 mm by checking if the remainder of a division from the diameter in mm by 10 is randomly distributed. For testing this hypothesis, a Chi-square test was applied where the observed distribution of the remainder values from 0 to 9 was compared with the expected equal distribution of remainder values. This analysis revealed that many observations do not have the accuracy of 1 mm, probably because traditional callipers typically have a 1 cm scale and finer measurements had to be estimated. Such estimates sometimes prefer 0 mm and 5 mm or even decimal places. The similar instrumental error was also expected for height measurements and thus we tested if the reading from height measurement had an accuracy of 1 dm by taking the remainder of a division from the height in dm by 10. It turned out that at least the distribution of the remainder of the given height value was in all cases close to a random distribution.

**Figure 3:**
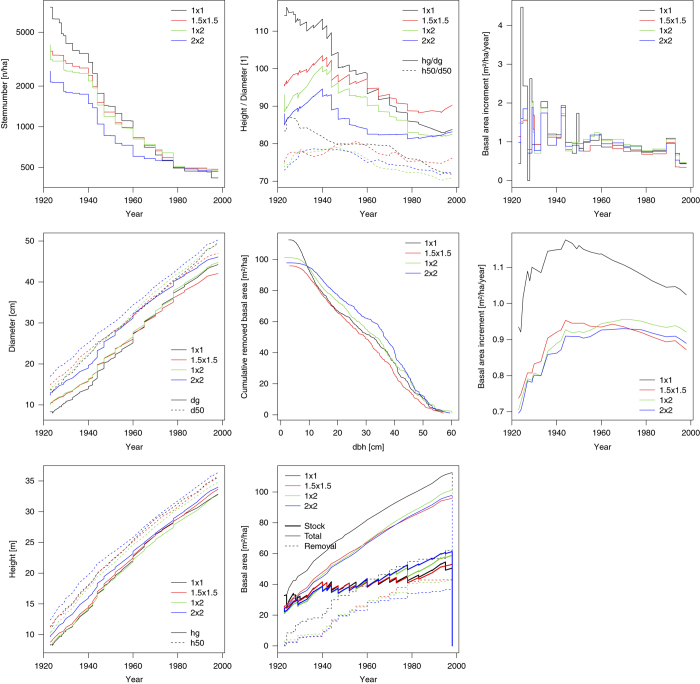
Characteristic plot development of stem number, diameter, height, height / diameter, cumulative harvests over diameter, basal area, periodical increments and mean annual increment. dg...Diameter of the stem with average basal area, d50...Diameter of the 50th strongest tree on one hectare, hg...Basal area weightened mean tree height, h50...Height of the 50th highest tree on one hectare.

The individual-tree measurements were aggregated and converted to hectare values and plotted to see if their development is plausible. Figure 3 shows the stem number development which is in 1925 on three plots close to the stem number at plantation. The average diameter is increasing from around 10 cm to 40 cm over the time. The visual steps in Fig. 3 arise when the removed trees have a different average diameter than the remaining trees. Tree height is increasing during the observation from 10 m to 30 m. The plot with the widest (2×2 m) spacing seems to have the best (h50=34.2 m at age 100 years), the plots with 1×1m (33.2 m) and 1.5×1.5 m (33.3 m) average and the plot with 1×2m the lowest (32.6 m) site index. The height to diameter ratio (H/D) is an indicator of stability. The cumulative harvested basal area over the diameter of the harvested tree is an indicator which assortments and their amount could be harvested. Also basal area stock, total increment and cumulative removals over the observation period are shown. Plot 1x1m has the highest basal area increment where this lead was achieved in young years by having there higher stocks. The periodical increment is fluctuating from period to period especially in very short periods, where the “noise” caused by measurement errors seems to be higher than the measured “signal”. The mean annual basal area increment is culminating for plot 1x1m around 1950 on a much higher level than the other 3 plots which are culminating around 1980. The observed values show plausible developments but existing random scatter is problematic for analyses depending on short time intervals.

## Additional information

**How to cite this article**: Kindermann, G. E. *et al.* 109 years of forest growth measurements from individual Norway spruce trees. *Sci. Data* 5:180077 doi: 10.1038/sdata.2018.77 (2018).

**Publisher’s note**: Springer Nature remains neutral with regard to jurisdictional claims in published maps and institutional affiliations.

## Supplementary Material



## Figures and Tables

**Table 1:** Size, initial spacing pattern and location of the four plots.

**Plot**	**Size**	**Spacing**	**Location**
1	82.2×61.1 m	1×1m	North-East
2	82.2×58.3 m	1.5×1.5 m	South-East
3	82.2×58.3 m	1×2m	South-West
4	82.2×61.1 m	2×2m	North-West

**Table 2:** Number of Observations.

**Plot**								**Type**				**1**	**2**	**3**	**4**	**1**	**2**	**3**	**4**	**1**	**2**	**3**	**4**	**1**	**2**	**3**	**4**	**1**	**2**	**3**	**4**
**Year**	**Obs**	**D.**	**H.**	**Trees**				**Diameter**				**Height**				**Crownheigth**				**Crownradius**						
1923	1	CC		1926	967	973	640	364	967	973	640	0	69	214	106	0	33	144	92	0	32	144	85
1924	1	CC		1926	898	759	532	347	898	759	532	0	13	8	0	0	12	0	0	0	0	0	0
1925	1	CC	Ba	1578	839	740	528	1579	839	740	528	1429	276	245	517	0	0	0	0	0	0	0	0
1927	1	CC		1576	839	740	528	1577	839	740	528	98	3	0	1	0	0	0	0	0	0	0	0
1928	1	CC		1475	0	0	0	1476	0	0	0	249	0	0	0	187	0	0	0	187	0	0	0
1929	1	CC		1223	836	740	527	1224	836	740	527	39	70	103	74	0	65	98	72	0	65	98	72
1929	2	CC		0	762	637	453	0	762	637	453	0	0	5	0	0	0	0	0	0	0	0	0
1930	1	CC		1184	762	632	453	1185	762	632	453	146	29	8	9	0	0	0	0	0	0	0	0
1933	1	CC		1038	728	624	443	1039	728	624	443	72	24	11	2	0	0	0	0	0	0	0	0
1936	1	CC	LT	919	694	610	440	920	694	610	440	108	118	127	62	84	102	116	54	83	102	117	55
1940	1	CC		875	673	600	433	876	673	600	433	112	71	68	64	53	51	62	63	53	51	62	63
1942	1	CC		753	595	527	369	131	102	59	26	131	102	59	26	52	59	34	19	52	59	34	19
1944	1	CC		622	493	468	343	623	493	468	343	118	110	102	77	57	70	72	64	57	69	72	64
1947	1	CC	LT	444	375	350	260	445	375	350	260	110	86	92	73	68	52	60	41	68	52	60	41
1949	1	CC		359	0	0	0	3	0	0	0	3	0	0	0	0	0	0	0	0	0	0	0
1950	1	CC		356	0	288	214	1	0	1	1	1	0	1	1	0	0	0	0	0	0	0	0
1952	1	CC	LT	355	318	287	213	356	318	287	213	75	91	88	63	71	90	87	62	71	90	87	62
1955	1	CC		315	265	253	187	37	20	16	6	37	20	16	6	27	20	16	6	0	0	0	6
1957	1	CC		0	0	237	0	0	0	1	0	0	0	1	0	0	0	0	0	0	0	0	0
1959	1	CC		278	0	236	0	1	0	2	0	1	0	2	0	0	0	1	0	0	0	0	0
1960	1	CC	LT	277	245	234	181	278	245	234	181	96	65	62	48	78	54	54	42	78	54	54	42
1965	1	CC	BLF	208	205	196	150	209	205	196	150	58	51	42	25	28	26	17	5	28	26	17	5
1970	1	CC	BLF	177	179	178	144	178	179	178	144	77	54	51	44	60	45	43	43	60	45	43	43
1973	1	CC	BLF	155	164	164	140	156	164	164	140	71	60	48	44	70	60	48	44	0	0	0	0
1978	1	Ta	BLW	142	146	155	139	143	146	155	139	69	61	58	62	69	61	58	62	0	0	0	0
1983	1	Ta	BLT	124	126	123	124	125	126	123	124	72	57	58	56	72	57	58	56	0	0	0	0
1989	1	Ta	Cl	118	121	120	121	118	121	120	121	66	53	55	54	66	53	55	54	0	0	0	0
1993	1	Ta	Cl	117	121	120	118	118	121	120	118	66	52	54	53	0	0	0	0	0	0	0	0
1995	1	Ta	Ve	116	120	117	117	116	120	117	117	115	120	116	117	100	120	114	116	0	0	0	0
1998	1	Ta	Ve	105	120	115	116	105	120	115	116	52	61	61	57	0	0	0	0	0	0	0	0
Obs..Observation in this year, D..Diameter, H..Height, Crownheight..Height to the crown base, CC..crosswise with calliper, Ta..girth with tapeline, Ba..bamboo poles, LT..ladder and tapeline, BLF..Blume-Leiss with fixed distance, BLW..Blume-Leiss variable distance after Johann (1974), BLT.. Blume-Leiss variable distance with tapeline, CL..Clinometer, Ve..Vertex																							
Note that in column “Trees” the number of trees, which are inside the polygon defined by the plot corners, are given. All other columns show the number of trees which have been measured and have been assigned to a specific plot.																							
